# Epidemiology of Tumor-Induced Osteomalacia in Denmark

**DOI:** 10.1007/s00223-021-00843-2

**Published:** 2021-04-05

**Authors:** Bo Abrahamsen, Christopher D. Smith, Salvatore Minisola

**Affiliations:** 1 Open Patient Data Explorative Network (OPEN), Department of Clinical Research, University of Southern Denmark and Odense University Hospital, 5000 Odense C, Denmark; 2 Department of Medicine, Holbæk Hospital, 4300 Holbæk, Denmark; 3 Nuffield Dept of Orthopaedics, Rheumatology and Musculoskeletal Sciences, University of Oxford, Oxford, OX2 6NN UK; 4 Department of Internal Medicine and Medical Disciplines, Sapienza University of Rome, Viale del Policlinico 155, 00161 Rome, Italy

**Keywords:** Tumor-induced osteomalacia, Hypophosphatemia, Phosphaturic mesenchymal tumors, Incidence, Prevalence, Epidemiology

## Abstract

Tumor-induced osteomalacia (TIO) is a rare, acquired condition of phosphate wasting due to phosphaturic mesenchymal tumors. Because the incidence and prevalence of TIO is unknown, we conducted an observational cohort study using national Danish health registers for the period 2008 to 2018 to obtain such information. The study also aimed to describe the demographics of the TIO population and the prognosis. The operational definition was based on hypophosphatemia or adult osteomalacia diagnoses, combined with prescriptions used in the initial management and procedures consistent with advanced imaging used for locating tumors. The incidence of TIO in Denmark was found to be below 0.13 per 100,000 person years for the total population of the country and 0.10 per 100,000 in adult-onset disease. The prevalence of TIO was estimated to be no more than 0.70 per 100,000 persons for the total population and 0.43 per 100,000 in adults. In 2018, there were a maximum of nine new cases of TIO in Danish adults. Mortality was low but few patients fulfilled the protocol cure criterion during the observation period. TIO has no ICD-10 code and limitations to the study include lack of information on serum biochemistry and on the use of phosphate supplements. Strengths include the use of long-term longitudinal, national hospital and prescription data from a country with universal healthcare. Given the very small patient population with TIO and the known delay to diagnosis and cure, management of patients with suspected TIO should be centralized.

**Supplementary Information:**

The online version contains supplementary material available at 10.1007/s00223-021-00843-2.

## Introduction

Tumor-induced osteomalacia (TIO) is a rare, acquired condition of phosphate wasting due to FGF23 secreting tumors [1]. Excess levels of FGF23 lead to chronic hypophosphatemia, which in turn can result in debilitating musculoskeletal deficits. The phosphaturic mesenchymal tumors (PMT) are in most cases very small and non-malignant. The tumor may secrete other phosphatonins in addition to FGF23. Surgical cure is possible in patients provided the location of the tumor can be accurately identified by imaging [2] and that the tumor can be reached and removed surgically [1, 3, 4], or through radiofrequency ablation [5]. Patients with TIO experience a sudden onset pronounced, often debilitating, myopathy of the proximal muscles, resulting at first in muscle pain and in a waddling gait and severe problems climbing stairs or rising from a chair. Bone pain and pathological fractures of the ribs, femur or pelvis are not uncommon if the diagnosis and management is delayed [1, 6]. In a Chinese case review of 144 cases of TIO [6], the clinical manifestations in decreasing order of prevalence were bone pain (in 99%), difficulty walking (93%), fracture (80%), height loss (69%) and muscle weakness (65%), followed by thoracic deformity, and tooth loss. The initial clinical suspicion is commonly lumbar disc herniation, spondylarthritis or osteoporosis. However, the condition may mimic diseases as diverse as motor neurone disease, polymyalgia, myositis, stroke or functional somatization disorder. Symptoms may be mistaken for simple vitamin D deficiency at first and the diagnosis is often delayed because serum phosphate may not be part of the routine biochemical assessment that patients receive.

Biochemically, patients with TIO typically present with an unusual combination of low serum phosphate, normal serum calcium levels, raised alkaline phosphatase and normal or elevated PTH. Measuring 1,25(OH)2D and finding it low further strengthens the suspicion of TIO but the essential step in the diagnosis is demonstrating inappropriately high renal excretion of phosphate, evident as a low TmP/GFR measured in a two hour urine collection in a fasting subject. Alternatively, %TRP can be measured. In a normal subject, TmP/GFR will be high in the presence of a low serum phosphate level whereas it is low in FGF23 driven hypophosphatemia including TIO [1].

Surgical removal of the tumor is the definitive treatment for TIO. Medical treatment to manage the chronic hypophosphatemia is required in the short-term management of patients up to the point of successful surgery and in the long term in patients where tumors cannot be identified and excised or eradicated by other means.

Despite the rise in the volume of publications on the pathophysiology and management of patients with TIO over recent years, there is a fundamental lack of knowledge about the true prevalence and incidence of TIO [7]. The challenge is amplified by the lack of a specific ICD-10 diagnosis code for TIO. Hence, detection of TIO patients in registers and databases will either require a very large manual review of case notes from a host of hospitals or, which is more cost effective, an epidemiology study using a combination of characteristic features based on a combination of ICD-10 and billing codes for the diagnostic work-up [1, 3] and treatment path [1] for these patients. There are no robust epidemiological studies on the incidence and prevalence of TIO. The only estimate in the literature comes from a survey sent out to Japanese hospitals [7]. Here the incidence of new TIO cases in Japan (population 127 million) was estimated to be roughly the same as newly diagnosed XLH cases. If correct, this would translate to 0.04 per 100,000 per year. It is not clear from the paper how selective sampling of specialist hospitals, their weighting to the results and the potential for the same patient to have been seen in more than one hospital—leading to double counting- was addressed in the study. In addition, it is unclear how sampling and calculation of incidence accounted for the difference in duration of the two diseases as XLH is a chronic condition whereas many cases of TIO are open to surgical cure.

Given the non-specific clinical presentation of the patients it must be expected that coding in routine data can change during the assessment of the patient. Hence, some patients who are initially coded with a broader ICD-10 code that is at first compatible with TIO will later be assigned a more specific code once they have completed their diagnostic workup. For example when the inherited phosphate wasting disorder XLH [8, 9] has been verified through genetic testing, TIO is then ruled out. An important opportunity to differentiate TIO from other phosphate wasting diseases in register data is that functional and anatomical imaging will be required to locate the FGF23-secreting tumor while this will not be the case for competing causes of hypophosphatemia [1, 2]. In cases open to surgical treatment there will be surgery and pathology encounters to confirm the case diagnosis. The absence of a code for surgery does not of course exclude TIO as some tumors cannot be removed. The clinical pathway of initial pharmaceutical therapy to treat the hypophosphatemia followed by the use of advanced imaging in order to plan surgical intervention was the rationale behind the case finding strategy, which is defined in the methods section below.

In order to address these gaps in knowledge, we conducted a register-based study on Danish national data to determine the incidence and prevalence of TIO in Denmark over a ten-year period and describe the demographics of TIO patients. The Danish health databases provide a unique opportunity to provide reliable estimates of TIO incidence, prevalence and prognosis.

## Methods and Study Population

### Objective

Describe the epidemiology of TIO in Denmark in terms of incidence, prevalence, demographics and prognosis using national registers.

### Analysis Strategy

The study was designed and reported in accordance with the STROBE guidelines for observational studies [10]. Based on the general medical strategy for management of TIO as reviewed elsewhere [1], our expectation was that the condition would be managed in the short term by using phosphate supplements and 1,25(OH)2D to increase serum phosphate to the lower end of the normal range and normalize serum alkaline phosphatase levels. The treating clinicians would attempt to localize the phosphaturic mesenchymal tumor by advanced imaging, using one or more of the imaging procedures listed below. Ultimately, most patients would be cured by surgery or radiotherapy. However, the analytic strategy had to take into account that no specific ICD-10 code exists for TIO and that there could be considerable variation in how the diagnosis was coded, especially if patients were seen in several clinics in the course of making the definite diagnosis. Hence the strategy had to throw out a relatively wide net of diagnoses and then refine the suspicion of TIO by tracking specific medications and specific imaging codes while ruling out competing indications for such exposures. Diagnosis codes for hospital contacts (please see below) are readily available for study as are prescriptions filled for one-alpha-hydroxylated vitamin D, and expensive procedures such as advanced imaging and tumor surgery will all result in procedure billing codes that could be accessed. By contrast, there is not a national resource from which laboratory results can be drawn to use serum phosphate or alkaline phosphatase levels to adjudicate the diagnosis. Further, in the development of the protocol we saw that phosphate supplements were not dispensed to patients through medications that were captured in the Danish Prescription Database. The study protocol was therefore based on identifying patients who met specific ICD-10 code definitions combined with one-alpha vitamin D treatment and advanced imaging, potentially also coupled with tumor surgery or radiotherapy.

### Study Design

Observational, open cohort study using national data from a country with universal healthcare.

### Study Population

Danish residents in the period 2007–2018, both genders, any age, with a sub-analysis done for adult-onset disease (age 18 or over). Only eligible for inclusion in incidence rate and prevalence calculations for calendar years that were preceded by a full calendar year of residence in the country. Observations were truncated when the subject was no longer resident in Denmark, at death or on the 31st of December 2018, whichever occurred first.

### Data Sources

The National Hospital Discharge Register (Landspatientregistret) contains all diagnostic codes and treatments for inpatients (since 1977 for public hospitals) and outpatients (since 1995 for public hospitals) [11]. It contains main diagnoses (“A diagnoses”) and up to twenty secondary diagnoses (“B diagnoses”) as ICD-10 codes, imaging- and surgical procedure codes (Danish administrative “SKS codes”), and admission/discharge dates. Treatment received at private hospitals, which constitutes a small proportion of the hospital activity in the country, is recorded from 2002 and onwards. Migration dates for entering and leaving Denmark are available through linkage with the National Civic Register so that years at risk can be determined accurately. The National Prescriptions Database (Lægemiddelregistret) [12] contains all prescriptions filled in the country since 1995. This register is the oldest national prescription registry in Europe and provides unique long-term information on drug exposure. The database is linked to the individual person by Danish social security number (assigned to all residents in Denmark at birth or immigration to the country) and contains a record of WHO Anatomic Therapeutic Chemical (ATC) code, date the drug was dispensed, tablet strength, and quantity dispensed. A repeat prescription (refill) generates a record in the database each time medications are dispensed from the pharmacy.

## Case Definitions (Fig. 1)

### Possible TIO (Protocol)

All persons with an ICD-10 encounter for *Hypophosphatemia* (ICD-10 E833A) or *Vitamin D resistant rickets* (E833B) or *Other adult osteomalacia* (M838), after exclusion of *XLH / Familial Hypophosphatemia* (E833A1). This is abbreviated group A in the results.

**Fig. 1:**
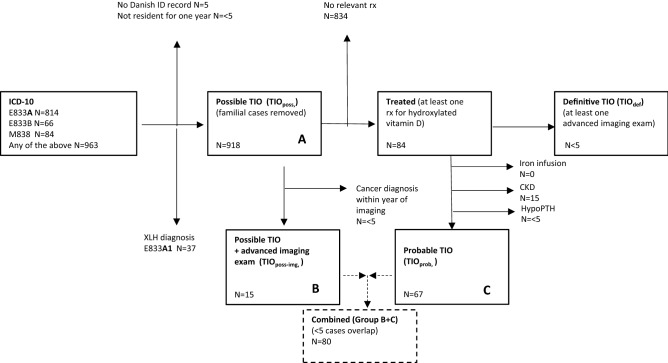
Flow chart

In addition, in a post hoc analysis we identified persons meeting this criterion who also had advanced imaging suggestive of TIO but had not filled prescriptions for one-alpha-hydroxylated vitamin D that would have placed them in the Definitive TIO category (see below). This addressed the possibility that patients could have received one-alpha-hydroxylated vitamin D by hand in outpatient clinics without requiring a prescription. We therefore created the additional operational definition “*Possible TIO with advanced imaging*”, abbreviated group B in the results). Following reviewer concerns we also investigated the presence of multiple myeloma—with the potential of hypophosphatemic osteomalacia—or drug induced adult-onset Fanconi syndromes.

### Probable TIO (Post Hoc)

Cases meeting the Possible TIO definition (see above) who also filled prescriptions for one-alpha-hydroxylated vitamin D (WHO ATC codes A11CC03, A11CC04), after exclusion of other indications. The case definition requires patients not to have consulted with CKD or hypoparathyroidism (± 1 year of the index date) or been treated with iron infusions. Abbreviated group C in the results.

### Definitive TIO (Protocol)

Cases meeting the Possible TIO definition and the advanced imaging criterion and who have filled prescriptions for one-alpha-hydroxylated vitamin D.

#### Incidence Rates

Incidence rates were calculated as the number of new cases with contact episodes within a calendar year meeting each of the operational definitions for TIO (see above), who did not have a contact episode for TIO in the preceding calendar year. We used 2007 as a run-in year to allow incidence rate calculations from 2008 and on. The denominator is the number of person years at risk (total population) in the calendar year. Rates were expressed as new cases per 100,000 person years.

#### Prevalence Proportion

Disease prevalence was calculated as the *period contact prevalence* with the numerator calculated as follows: The number of persons with at least one contact episode for TIO (each operational definition) in the calendar year. Denominator: Number of persons at risk (equivalent to person years at risk in a one-year contact prevalence scenario). Prevalence was expressed as cases per 100,000 population.

#### Relative Risk Estimates

Not applicable.

#### Missing Data

Patients lacking a Danish ID number were not included in the analysis (*N* = 5, Fig. 1) because age and gender information as well as links to prescription information needed for the operational case definitions would be unavailable. No imputation was done. For the purpose of this analysis, the information held in the national prescription register and national patient register were considered a true record of contacts since the absence of hospital contacts and filled prescriptions in a given time period does not in itself indicate missing data but is what will be seen in many younger people in good health.

#### Ethics, Patient Privacy and Support

The researchers did not have access to information that would allow them to directly or indirectly identify the persons in the study. Statistics Denmark receive person level health data and demographic information from the Danish authorities including the Health Data Board and the Danish Medicines Agency and can grant approved research institutions access to de-identified microdata, i.e. linked person level data where the social security number has been replaced by a bogus ID that is consistent across registers. The corresponding author had full access to all of the data in the study and had final responsibility for the decision to submit for publication.

## Results

A total of 918 cases (Fig. 1) of possible TIO were identified as consulting in the time period 2008–2018, after removal of 5 cases with no linkable Danish ID record and 3 that had not been resident in the country for one year, and 37 cases that were coded as familial hypophosphatemia (ICD-10 code E833A1). The total follow-up time for the 918 persons enrolled in the analysis was 2,491 person years from diagnosis index date ('Possible TIO'), with a median follow-up time of 1.7 years (range 0.0 to 11.0 years) and a mean follow-up time of 2.7 years (SD 2.7). Competing causes and differential diagnoses: Of the 918 persons with a case definition of Possible TIO, one had a history of adult onset Fanconi syndrome and five had a history of multiple myeloma, which are potential competing causes of hypophosphatemia. At most, this would cause less than a 1% overestimation of TIO cases in the following. However, of these six cases met the subsequent criteria (Probable or Definitive TIO) or received advanced imaging.

### Incidence of TIO

**Fig. 2:**
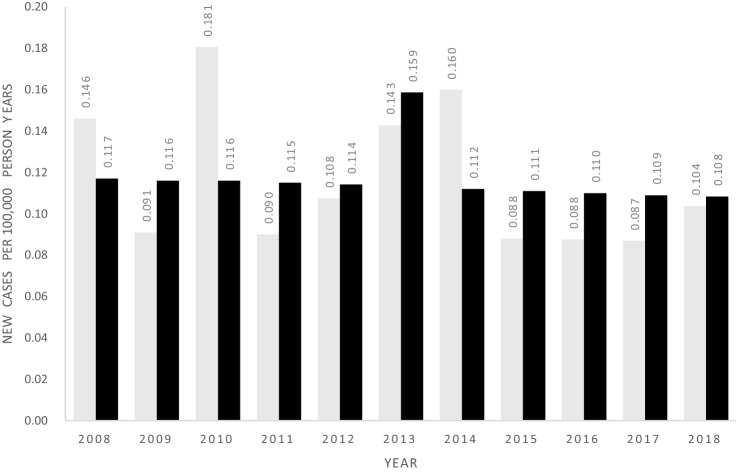
Incidence per 100,000 person years of Probable TIO in Denmark after removal of cases with CKD, hypoparathyroidism or iron infusion. Childhood onset and adult onset combined (grey bars) vs adult onset only (black bars)

Fewer than five persons (< 0.008 cases per 100,000) met the protocol case definition of definitive TIO, i.e. the required combination of diagnosis code, advanced imaging and prescriptions filled for one-alpha-hydroxylated vitamin D. Statistics Denmark rules for data privacy do not permit analysis of groups with fewer than five members so additional details cannot be presented for this subcategory. The study identified 15 cases of possible TIO who also underwent advanced imaging, corresponding to 0.024 per 100,000 patient years. The imaging used was F-18-FDG in 14 cases and Ga-68-DOTATOC in one case. A further 67 cases met the definition of probable TIO after exclusion of CKD and iron infusion as competing causes (Fig. 1), corresponding to 0.11 cases per 100,000 patient years (Table 1 and Fig. 2). The combined incidence of these two categories, after removing overlapping cases, was 80 persons or 0.13 per 100,000 patient years, i.e. about eight new cases per year in the country.

**Table 1:** N and Incidence rate per 100,000 by operational group, annually and across the period of study

Year	Pop at risk	A “Possible TIO”	B "Possible TIO" with advanced imaging	C. "Probable TIO" after removal of CKD and iron cases	Group B + C	A “Possible TIO”	B "Possible TIO" with advanced imaging	C "Probable TIO" after removal of CKD and iron cases	Group B + C
2008	5,475,791	22	< 5	8	8	0.402	< 0.091	0.146	0.146
2009	5,511,451	29	< 5	< 5	< 5	0.526	< 0.091	< 0.091	< 0.091
2010	5,534,738	28	< 5	10	10	0.506	< 0.09	0.181	0.181
2011	5,560,628	43	< 5	< 5	< 5	0.773	< 0.09	< 0.09	< 0.09
2012	5,580,516	56	< 5	6	6	1.003	< 0.09	0.108	0.108
2013	5,602,628	69	< 5	8	8	1.232	< 0.089	0.143	0.143
2014	5,627,235	101	< 5	9	11	1.795	< 0.089	0.160	0.195
2015	5,659,715	98	< 5	< 5	6	1.732	< 0.088	< 0.088	0.106
2016	5,707,251	153	< 5	5	6	2.681	< 0.088	0.088	0.105
2017	5,748,769	159	< 5	< 5	7	2.766	< 0.087	< 0.087	0.122
2018	5,781,190	160	5	6	10	2.768	0.086	0.104	0.173
2008 to 2018	61,789,912	918	15	67	80	1.486	0.024	0.108	0.129

Total study population irrespective of age at onset

#### Adult-Onset TIO

If restricting the analysis to subjects with adult-onset disease, the incidence rate of *Possible TIO with advanced imaging* was 0.027 per 100,000 patient years, while the incidence rate of *Probable TIO with exclusion of competing causes* was 0.076 per 100,000 patient years and the combined incidence of the two was 0.10 per 100,000 patient years.

### Prevalence of TIO

The *contact prevalence* (persons consulting) varied over the duration of the study with 2015 having the highest contact prevalence for *probable TIO* (Suppl Fig. 1) and 2018 the highest contact prevalence for *possible TIO with advanced imaging* (data not shown). For the most recent year, 2018, there were 27 patients—17 women and 10 men—still consulting after either possible TIO with advanced imaging (*N* = 7) or the case definition of probable TIO (*N* = 22 as categories overlap), corresponding to a combined contact prevalence of 0.47 per 100,000 persons.

In the sub analysis restricted to subjects aged 18 or over, there were 15 cases consulting in 2018 after either a possible TIO with advanced imaging (*N* = 6) or a probable TIO after excluding competing causes (*N* = 10), for a combined contact prevalence of 0.325 per 100,000 persons.

**Fig. 3:**
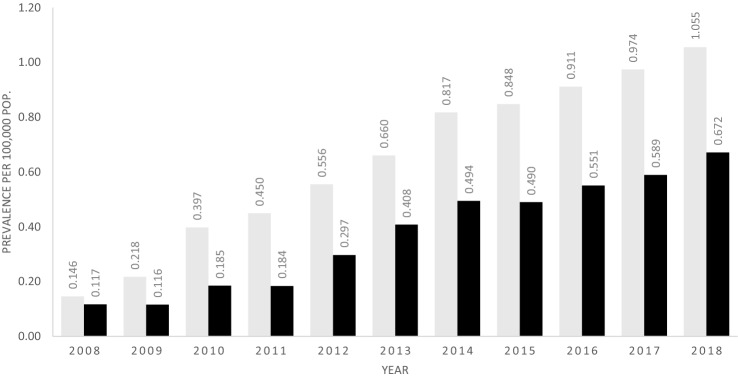
Prevalence per 100,000 persons of Probable TIO in Denmark after removal of cases with CKD, hypoparathyroidism or iron infusion. Childhood onset and adult onset combined (grey bars) vs adult onset only (black bars)

The *population prevalence* includes all patients who remained alive and had no Danish healthcare record of having undergone curative radiotherapy or surgery. Population prevalence increased over the study period (Fig. 3). The total number of persons alive in Denmark in 2018 who met this criterion was 72 for possible TIO with imaging and probable TIO combined, or 1.25 per 100,000.

#### Adult-Onset TIO

In 2018, there were 41 persons alive with onset at age 18 or over who met the criterion for *possible TIO with imaging* and/or *probable TIO after excluding competing causes*, giving a maximum population prevalence of 0.89 per 100,000 persons.

### Demographics for TIO at the Time of Diagnosis (Table 2 and 3)

The demographics differed considerably between the Possible TIO with advanced imaging category, where 60% of patients were men and the mean age was 61.8 years, and the Probable TIO category which had two thirds women and a much younger mean age of 32.1 years. The age range was considerable in both groups, however, with the youngest patients being 14 and less than a year old while the oldest were 89 and 97.8 years old, respectively. The diagnoses coded at the initial presentation for the total population and the adult population specifically, are shown in table XE for reference.

**Table 2:** Demographic characteristics by operational definition

Case definition	A “Possible TIO”	B "Possible TIO" with advanced imaging	C "Probable TIO" after removal of CKD and iron cases	Group B + C
*N*	918	15	67	80
Age (SD, Range)	58.2 (25.0, 0–101)	61.8 (25.1, 14–89)	32.1 (26.9, 0–98)	37.9 (29.1, 0–98)
Men	43.2%	60%	34.3%	40.0%
Women	56.7%	40%	65.7%	60.0%
Country of origin				
Denmark	93.7%	100.0%	98.5%	98.8%
Western Europe	2.5%	< 5	< 5	< 5
Rest of world	3.8%	< 5	< 5	< 5
Composition of ICD-10 groups				
Hypophosphatemia	84.6%	93.3%	38.8%	48.8%
Vitamin D resistant rickets	6.2%	< 5	52.2%	43.8%
Other adult osteomalacias	9.2%	< 5	9.0%	7.5%

**Table 3:** Diagnosis codes at presentation, by operational definition, total vs adult onset population

Case definition	ICD-10 at presentation	Any age	Adult onset (age 18 and over)
B "Possible TIO" with advanced imaging	E833A Hypophosphatemia	93.3%	92.3%
	E833B Vitamin D resistant rickets	< 5	0.0%
	M838 Other adult osteomalacias	< 5	< 5
C "Probable TIO" after removal of CKD and iron cases	E833A Hypophosphatemia	38.8%	40.5%
	E833B Vitamin D resistant rickets	52.2%	43.2%
	M838 Other adult osteomalacias	9.0%	16.2%
Group B + C	E833A Hypophosphatemia	48.8%	55.1%
	E833B Vitamin D resistant rickets	43.8%	32.7%
	M838 Other adult osteomalacias	7.5%	12.2%

#### Prognosis

We considered patients cured of TIO once a record had been made of them undergoing tumor surgery or radiotherapy in Denmark and no longer filling prescriptions for alpha-hydroxylated vitamin D treatment. The patient flow into one-alpha-hydroxylated vitamin D treatment, radiotherapy and surgery is summarized below (Table 4). Even when combining groups B and C, the total number of patients receiving radiotherapy or tumor surgery was below the reporting threshold of five cases. Survival prospects were very good with only 8 deaths among the 80 cases with *Possible TIO with advanced imaging* or *Probable TIO*, corresponding to a mortality rate of only 1% per year. Given the low number of deaths we did not calculate a standardized mortality rate as it would have a wide confidence interval.

**Table 4:** Treatment, cure and mortality by TIO operational definition

	B "Possible TIO" with advanced imaging	C "Probable TIO" after removal of CKD and iron cases	Group B + C
Total N with condition	15	67	80
Treatment/workup			
One-alpha-hydroxylated vitamin D	< 5	67	67
Advanced imaging	15	< 5	15
Radiotherapy	< 5	< 5	< 5
Surgery	< 5	< 5	< 5
Radiotherapy or surgery	< 5	< 5	< 5
Cure criterion met	< 5	< 5	< 5
Died	< 5	6	8
Mortality rate (% per year)	< 3.1%	0.9%	1.0%

## Discussion

This study confirms that the incidence and prevalence of TIO in Denmark is very low, even if applying upper boundary estimates that include patients who may have TIO but do not meet the operational case definition in full. In brief, the prevalence of *'Definitive TIO'* over the eleven years covered by the study (2008 to 2018) was under five cases—the lowest number for which we are permitted to present data under Statistics Denmark regulations—corresponding to a prevalence below 0.008 per 100,000. A more detailed analysis including upper boundary estimates—advanced imaging and/or one-alpha-hydroxylated vitamin D without a competing indication—found an incidence rate of 0.17 per 100,000 for 2018 and 0.13 for the full study period, with a 2018 upper boundary prevalence of 1.25 per 100,000 and a contact prevalence of 0.47 per 100,000. The incidence rates found increased relatively little over time with 2018—about 25% from 2008 to 2019—but prevalence increased due to low mortality and few recorded instances of tumor surgery or radiotherapy in Denmark for this population.

The low number of cured cases in the present report may seem at first surprising given the theoretically reversible nature of this condition. However, findings should be viewed in the context that cohort inclusion over a 10-year period means that half the cases have less than 5 years of follow-up after diagnosis. The reported US experience [14–16] is that the average time from diagnosis of TIO to successful localization of the tumor is 5 years so many TIO cases in this report may still be in the process of having their tumor located and removed. Moreover, a recent report [13] covering 230 patients with TIO found that 11% of cases persisted after primary surgery and that 7% of cases recurred, with the refractory rate being as high as 78% for PMT located in the spine. Further, it remains possible that a small number of patients with PMT in surgically challenging locations may have received gamma knife procedures or other advanced surgery at cancer centers outside the country after referral by the Danish National Health service and this is not traceable in our data sources. Limitations to our study include lack of data on serum phosphate levels, on any confirmatory FGF23 measurements and on use of phosphate supplements which could have provided clearer guidance to verify the diagnosis. Further, adult onset secondary Fanconi syndrome and myeloma with hypophosphatemia are additional differential diagnoses that were not considered in the protocol; however fewer than 1% of cases in the study had a history of these conditions and none received advanced imaging or met the study criteria for Probable or Definite TIO.

We noted that an unexpectedly high proportion of potential cases were under the age of 18 at the time of meeting the operational diagnosis definition. Though TIO has been reported in children as young as 3 years of age [17, 18], this is generally a disease that develops in adults. Very early onset of a requirement for one-alpha-hydroxylated vitamin D in the absence of parathyroid or renal disease is more suggestive of a genetic disease such as XLH or VDDR.

The adult onset upper boundary estimator found an incidence rate of 0.10 per 100,000 person years for the full study period, a population prevalence of 0.43 per 100,000 persons and a contact prevalence of 0.25 per 100,000 persons (Summary in Table 5). In 2018 there were nine new adult patients meeting the combined operational criterion and a total of 15 cases consulting in Denmark.

**Table 5:** Summary of incidence and prevalence of TIO in Denmark 2008 to 2018

Case definition	Metric	Any age	Adult onset (age 18 and over)
B "Possible TIO" with advanced imaging	Incidence	0.024	0.027
	Population prevalence	0.068	0.074
	Contact prevalence	0.044	0.047
C "Probable TIO" after removal of CKD and iron cases	Incidence	0.108	0.076
	Population prevalence	0.644	0.372
	Contact prevalence	0.434	0.220
Group B + C upper boundary estimator	Incidence	0.129	0.101
	Population prevalence	0.698	0.430
	Contact prevalence	0.463	0.250

Excluding persons under the age of 18 from the incidence and prevalence calculations primarily affected subjects whose initial diagnosis was Vitamin D Resistant Rickets. This condition generally clinically manifests in childhood but we included it in the diagnoses to capture for case finding because it is accompanied by significant hypophosphatemia and could be the initial suspicion at referral and may be used by non-expert clinicians given the rachitic phenotype, low phosphate and requirement for one-alpha-hydroxylated vitamin D. However, in children TIO is an unlikely differential diagnosis as discussed above.

Irrespective of subpopulation, the incidence rates were found to increase relatively little over time with 2018—about 25% from 2008 to 2019—but the prevalence increased due to low mortality and few recorded instances of tumor surgery or radiotherapy in Denmark for this population.

In conclusion, in this study—which is the first register-based epidemiological study of TIO—the incidence in Denmark for the period 2008–2018 can be estimated as being below 0.13 per 100,000 person years for the total population of the country and 0.10 per 100,000 in adults. The study protocol pre-specified a total population assessment but due to the low likelihood of TIO in children and the dominance of genetic rather than acquired causes of hypophosphatemia in this age group, we consider the adult onset the more reliable assessment of the true incidence of TIO in the country. The prevalence of TIO was estimated to be no more than 0.70 per 100,000 persons for the total population and only 0.43 per 100,000 persons in adults. In plain numbers, in 2018 there were a maximum of nine new cases of TIO in Danish adults and up to fifteen persons consulting.

Given the very small patient population with TIO and the considerable delay to diagnosis and cure, management of patients with suspected TIO should be centralized. In order to facilitate referral and at the same time provide a better understanding of the epidemiology of TIO globally, in our opinion the condition should be given a distinct ICD-10 diagnosis code to differentiate it from other adult osteomalacias and hypophosphatemias. The importance of more widespread use of serum phosphate measurement in patients with non-specific muscle or bone symptoms should be emphasized as should increasing the availability of FGF23 measurement to help close the diagnosis and treatment gap for this disease.

Directions for future research include detailed, population-based review of individual medical notes and serum biochemistry of possible TIO cases and/or establishment of linkage between large clinical biochemistry, pharmacy and administrative hospital data to further narrow down the estimated incidence and prevalence.

## Supplementary Information

Below is the link to the electronic supplementary material.

Supplementary file1 (DOCX 38 kb)

## Data Availability

Danish law does not allow investigators to share data accessed through Statistics Denmark.
