# Supplementary material for: Epidemiology of Tumor-Induced Osteomalacia in Denmark
Source: Calcif Tissue Int. 2021 Apr 5;109(2):147–56. doi: 10.1007/s00223-021-00843-2 (PMC8273058; doi:10.1007/s00223-021-00843-2)
Supplement: Supplementary file 1 — Supplementary file1 (DOCX 38 kb) [file 223_2021_843_MOESM1_ESM.docx]

Supplementary Material

|  | SKS code |
| --- | --- |
| Whole body scan, F-18-FDG | WDLPSFAXX |
| Whole body scintigraphy, Tc-99m-MIBI | WDTGS20XX |
| Whole body scintigraphy, In-111-Octreotide | WDTGSI2XX |
| Whole body scintigraphy, I-123-MIBG | WDTGSJHXX |
| Tumor scan, Cu-64-DOTATATE | WDTPSCUXX |
| Tumor scan, F-18-FDG | WDTPSFAXX |
| Tumor scan, Ga-68-Octreotid | WDTPSG1XX |
| Tumor scan, Ga-68-DOTATATE | WDTPSG2XX |
| Tumor scan, Ga-68-DOTANOC | WDTPSG3XX |
| Tumor scan, Ga-68-DOTATOC | WDTPSG4XX |
| Whole body scintigraphy, SPECT, In-111-Octreotide | WDTSSI2XX |
| Whole body scintigraphy, SPECT, In-111-Octreotide | WDTSSI2XX |
| Whole body scintigraphy, SPECT, I-123-MIBG | WDTSSJHXX |
| Whole body scintigraphy, SPECT, I-123-MIBG | WDTSSJHXX |

**Supplementary Table 1: Hospital imaging procedures and SKS procedure codes considered consistent with TIO localization imaging in the study.**

**Figure S1:** Contact prevalence per 100,000 persons of Probable TIO in Denmark after removal of cases with CKD, hypoparathyroidism or iron infusion. Childhood onset and adult onset combined (grey bars) vs adult onset only (black bars).
